# Strengthening malaria diagnosis and appropriate treatment in Namibia: a test of case management training interventions in Kavango Region

**DOI:** 10.1186/1475-2875-13-508

**Published:** 2014-12-18

**Authors:** Christopher Lourenço, Deepika Kandula, Leena Haidula, Abigail Ward, Justin M Cohen

**Affiliations:** Clinton Health Access Initiative, Boston, MA 02127 USA; National Vector-borne Diseases Control Programme, Ministry of Health and Social Services, Windhoek, Namibia

## Abstract

**Background:**

Despite its importance in control and elimination settings, malaria diagnosis rates tend to be low in many African countries. An operational research pilot was conducted in Namibia to identify the key barriers to appropriate diagnosis of malaria in public health facilities and to evaluate the effectiveness of various training approaches in improving the uptake and adherence to rapid diagnostic tests (RDTs).

**Methods:**

After identifying case management weaknesses through focus group discussions, training interventions were designed to address these barriers over a six-month period. The study had three intervention districts and one control within the Kavango region of Namibia where poor case management practices were observed. The interventions included an enhanced training model, clinical mentorship, and SMS reminders. Monthly data on testing and treatment were collected for the period of April to September 2012 and, for comparison, the same months during the prior year from all 52 health facilities in Kavango. The same indicators were also obtained at district level for a follow-up period of 15 months from October 2012 to December 2013 to observe whether any improvements were sustained over time.

**Results:**

All intervention arms produced significant improvements in case management practices compared to the control district (all p < 0.02). Overall, districts receiving any training improved testing rates from 25% to 66% at minimum compared to the control. The enhanced training plus mentorship arm resulted in a significantly greater proportion of fevers receiving RDTs compared to the district receiving enhanced training alone, increasing from 27% to over 90% at endline. No ACT was prescribed to untested patients after caregivers received mentorship or SMS reminders. These improvements were all sustained over the 15-month follow-up.

**Conclusions:**

These changes show a reversal of improper case management practices over the six-month study period and demonstrate that implementing simple training interventions can have a significant, sustainable impact on the uptake of and adherence to malaria RDTs. Findings from this work have already informed Namibia’s roll out of a more robust case management training programme. The approaches used in Namibia may be applicable to other resource-constrained countries, providing practical guidance on sustainable approaches to febrile illness management.

**Electronic supplementary material:**

The online version of this article (doi:10.1186/1475-2875-13-508) contains supplementary material, which is available to authorized users.

## Background

Accurate malaria diagnosis is essential to know where transmission is occurring and to optimize design and targeting of interventions. Malaria diagnosis based on clinical signs and symptoms alone tends to result in substantial overdiagnosis of malaria, leading to wastage of resources and failure to treat the true causes of patients’ illnesses [1, 2]. Parasitological confirmation of malaria with rapid diagnostic tests (RDTs), microscopy, or molecular methods is particularly critical for countries approaching malaria elimination since a smaller proportion of fevers will be attributable to malaria, and understanding where transmission is occurring is a prerequisite to eliminating the final malaria foci.

Despite their importance, testing rates tend to be quite poor in the World Health Organization (WHO) Africa Region, as indicated by a 2014 13-country meta-analysis confirmation rate of 16% [3]. Experiences from other countries suggest that availability of diagnostic tools alone may be insufficient to improve case management practices [4] and that the simple dissemination of written guidelines is often ineffective [5, 6]. Additional supportive interventions, such as basic training packages and the use of clinical algorithms have been attempted to improve use of diagnostics and adherence to test results, but achieving sustained improvements has proved challenging [7, 8]. Assessments have identified deficiencies in the design and implementation of training models [5, 9], with inadequate curriculum content, insufficient supervision of trainees, lack of integration of trainee values and opinions [8], and underutilization of opportunities for contextual and collaborative learning [10].

Successful interventions to improve healthcare worker (HCW) practices have tended to involve consistent supervision and dissemination of feedback, and multifaceted interventions appear to have greater impact than single interventions [5, 11, 12, 9]. Participatory learning techniques and follow up support have proven to be effective methodologies across a variety of disease contexts and health settings [5, 13], and participatory group workshops were shown to be most effective when trainees interacted with their peers [14–17]. Mobile devices used for distance learning and SMS reminders have exhibited broad impact across HIV/AIDS [18, 19] and malaria training [20, 21] in resource-limited settings such as South America and southeast Africa. These methods can be quite cost-effective if the relevant technological capabilities are available in-country, such as a developed telecommunication network [22]. Case supervision as a form of training has also shown consistently positive effects on trainee performance, regardless of health cadre or patient population treated [16, 5]. Supervision allows for greater dialogue between trainer and trainee compared with didactic teaching and facilitates the adaptation of techniques to meet the needs of diverse learners [5].

Confirmed diagnosis rates remain low in the southern African country of Namibia, which is aiming for malaria elimination by 2020, and needs to achieve 100% parasitological diagnosis before the pre-elimination shift in 2016 [23]. In 2011, an estimated 60% of malaria cases reported by public health facilities were parasitologically confirmed nationally, with rates varying from 30% to 90% across regions [23]. While recent training sessions conducted by the National Vector-borne Diseases Control Programme (NVDCP) were aligned with current WHO standards, no consistent methodology had been implemented. In addition, a health facility census conducted in 2009 estimated only 12% of HCWs had received training on malaria diagnosis and treatment in the previous 12 months [24]. This gap may be partly attributed to the rapid turnover of health staff as well as the training of trainers (TOT) model which failed to cascade critical knowledge and skills from the top down. As stated during pre-study focus group discussions, HCWs explained the TOTs did not include any instruction or supportive tools for in-service training once trainees returned to their respective facilities. Accordingly, this study sought to identify barriers to appropriate malaria case management and assess whether improvements to the existing training programme could strengthen diagnosis and treatment practices in Namibia.

## Methods

### Intervention design

Focus group discussions (FGDs) and key informant interviews were used to elucidate weaknesses of the current case management programme. Clinical diagnosis of malaria has been common practice in Namibia for many years and discussions with nurses and their supervisors revealed a distrust of RDT reliability and their ability to use them. Ninety percent of FGD participants believed that the TOT model was the most appropriate training approach due to logistical restraints (e.g. travel time/distance, high workload). However, HCWs strongly expressed the need for training on malaria case management to be accompanied by supportive materials such as wall charts or other job aids that highlighted current malaria case management guidelines. Ninety percent of HCWs said they felt more comfortable making reference to a flow chart or algorithm in front of patients, as opposed to searching through a manual, because it was quick and there was already a level of patient familiarity with these types of charts for other disease groups. Finally, the FGDs also suggested that follow-up support was lacking, and some form of supervision, reminders, and/or refresher training may improve retention of key information and maintenance of skills.

An enhanced TOT model incorporating a revised curriculum was designed to address the barriers identified by the FGDs. Grow Training and Advisory Services CC, a Namibia-based consultancy that applies general adult learning and appreciative inquiry principles and approaches to business training, was engaged to support the NVDCP on developing the enhanced TOT curriculum, materials, and structure of the training. The TOT consisted of an interactive curriculum, participatory RDT practice sessions, mock cases, and a pre and post training skills assessment (see Additional file 1 for full list of modules and new training activities). The training also included a newly developed module on differential diagnosis to help HCWs manage RDT negative cases according to national guidelines. This module included a review of the decline in malaria burden and the most prevalent causes of fever in Namibia to reinforce the growing need for accurate diagnosis of non-malarial fevers. The training agenda was restructured to achieve greater efficiency by covering key topics over two to three working days compared to the original TOT which ran for five days. Six “Selected to Achieve Results” (STAR) facilitators, two per intervention district, were chosen to participate in an initial, comprehensive training session to learn the enhanced TOT format and to master teaching methods in preparation for the TOTs they would lead in their respective districts. After completion of the district-level TOT, HCWs went back to their health centres and clinics to conduct in-service training. To ensure all trainers were providing consistent information to their colleagues during in-service training, each trainer received a package of supportive materials including training manuals, standard operation procedures, and quick reference sheets on RDT use, diagnostic wall charts/algorithms for malaria and differential diagnosis, and evaluation forms. The research staff made multiple visits to random health facilities in the intervention districts during the second month of data collection to monitor in-service training completion.

In addition to the enhanced TOT, two supporting interventions were designed. The first, onsite mentorship, aimed to overcome the challenge of limited human resource capacity and nurses not being able to leave facilities for extensive training sessions [17]. Such mentorship has been shown to improve HCW knowledge and confidence in skills by facilitating greater dialogue between trainer and trainee compared with didactic teaching [25]. Two senior HCWs who were more experienced, well respected among their peers, and could effectively guide the professional development of HCWs were identified for the mentoring role. Each mentor, designated as a mobile malaria mentor (MMM), was assigned 3–5 health facilities, with priority given to rural clinics where no doctors or pharmacists were available onsite. MMMs observed the performance of HCWs using simple checklists to measure performance and track major issues and then shared feedback with the HCW as an entry point for discussion and refresher training on targeted areas for improvement. MMMs also conducted rapid competency assessments such as asking HCWs to interpret pictures of RDT results or asking basic questions on RDT technique (i.e., how many drops of buffer is required for each test strip, how long do you wait before reading the test results) and reviewed cases with HCWs, a learning technique that asks the HCW to talk through and review both RDT positive and negative cases and how these patients were managed. Follow-up visits to HCWs were made to measure improvement and to conduct a close-out session with health facility supervisors to review the issues covered during mentorship.

The second supportive intervention involved an SMS reminder system. Namibia has a highly developed mobile network and Make the Connection (MTC), the mobile provider with over 90% of the market share, was engaged to implement this intervention. The NVDCP designed educational SMS messages that were sent to HCWs in the target areas on a daily basis for two months. Agreements were arranged between the Ministry of Health and Social Services and MTC for the authorization and set up of the SMS server portal to direct the reminders via an automated system. A total of 11 messages were rotated over the two month period, and included short instructive phrases reminding HCWs on proper malaria diagnostic practices, RDT techniques/quality control checks, differential diagnosis, and information to share with the community with respect to malaria prevention. For example, “*Remember that ONLY TWO DROPS of buffer are used with the CareStart Combo RDTs*”.

### Site selection

The research pilot was conducted in four districts in the endemic northern region of Kavango, Namibia. Three of the districts were each assigned a different intervention mix. Nankudu district received the enhanced TOT alone, Andara district received the enhanced TOT supplemented by the mentorship intervention, and Rundu district received the enhanced TOT with SMS follow-up reminders. The fourth district, Nyangana, received no training intervention and served as a control. Control versus intervention selection was not random; Nyangana was known to have better testing and appropriate treatment rates at baseline and thus intervention was preferentially targeted elsewhere.

### Intervention launch and data collection

Letters were sent from the NVDCP to the Regional Health Director of Kavango explaining the national directive of the research study and outlining the protocol and selection of districts for the control and intervention arms. The district level TOTs conducted by the pairs of lead facilitators began in April 2012 and supporting interventions were initiated in May 2012. All public health facility workers in the intervention districts were eligible for participation in the study. Other cadres of health workers, such as medical officers or pharmacists were not blocked from training attendance, although preference was made to all nurses responsible for malaria case management. Clinics with only one staff were asked to send their nurse if an adequate substitute could be made during the district training session. HCWs at private health facilities were not included in this study.

Monthly data on the number of febrile malaria patients seen at the facility, the number tested and not tested by RDT, and the number who received an ACT following a positive test result, a negative result, or without a test were collected from facility registries for the intervention period of April to September 2012 and, for comparison, the same months during the prior year from all 44 health facilities in the intervention districts. Microscopy data were not included since the majority of health facilities use RDTs, while only the 4 disitrct hospitals have laboratory capabilities. Paper records were double entered electronically and stored centrally at the NVDCP office. Out-patient registry review took place either at the district hospitals or at site at each health facility. Quality assurance rounds were conducted during data collection at randomly selected sites to ensure proper completion of all forms. The same indicators were also collected in aggregate at district level through weekly surveillance data sent to the NVDCP for a follow-up period of an additional 15 months from October 2012 to December 2013 to assess whether any improvements were sustained over time.

### Data analysis

Testing and treatment rates in months following intervention implementation were compared to rates for the same months in the prior year, and the significance of these changes in intervention districts relative to those in the control district was assessed with repeated measures binomial regression models using the GENMOD procedure in SAS v9.3. The outcome for these models was the monthly number of tests conducted out of the total number of fevers observed (or in other models, other fractions such as the number of ACT prescribed following a positive test out of the total number prescribed) and predictor variables included an indicator variable representing whether or not a health facility was in an intervention district, a binary variable indicating whether the month occurred before or after initiation of interventions, and the interaction term between these variables to indicate whether pre-post changes were significantly greater in intervention health facilities compared to control facilities. An exchangeable correlation structure was used to account for repeated measures from the same health facility. To adjust for potential confounders, all models included covariates related to health facility type (hospital, health centre, or clinic); whether or not the facility was run by the government; the surrounding population density [26]; the accessibility to cities of at least 50,000 people; and, as a rough correlate for malaria transmission suitability, the elevation. Finally, to examine whether supporting interventions caused changes beyond those of the TOT alone, three additional variables were added: an indicator variable representing whether or not a health facility was in a district receiving that supporting intervention, a binary variable indicating whether the month occurred before or after initiation of the supporting intervention, and the interaction term between these variables to indicate whether pre-post changes were significantly greater in health facilities receiving supporting interventions compared to all other facilities.

## Results

**Figure 1:**
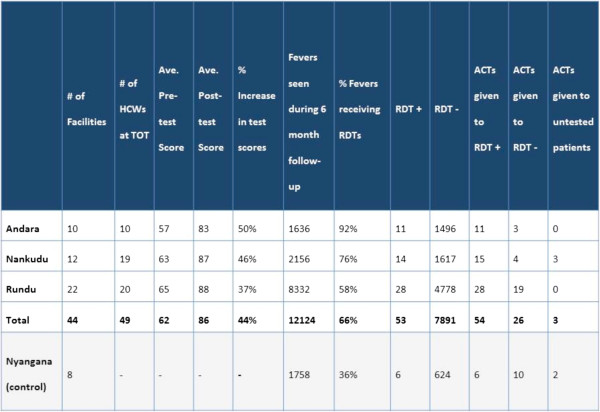
**District level indicators post-follow-up period.**

In total, six district facilitators were trained in the intervention region, 56 HCWs at the district workshops, and at least 100 total HCWs were reached at the in-service health facility level. These totals exceeded the study’s initial target reach. Substantial improvement in test scores was observed in all three intervention districts after training (Figure 1) and 89% of HCWs rated the training workshops very favourably (either *excellent* or *very good*) in the evaluations.

### Testing rates

A total of 12,124 fevers were reported in health facilities in the three intervention districts during the six months after implementation of the training intervention, compared with 18,703 during the same six month period in the prior year. Of these, 7,944 (65.5%) were recorded to have received an RDT after initiation of training, compared to 4,663 (24.9%) in the prior year. Monthly testing of febrile cases increased from a health facility mean of 31.2% to 76.3% following implementation across the three intervention districts, a highly significant improvement compared with the control district, where mean monthly facility testing rates declined from 50.8% to 41.8% (Z = 4.71, p < 0.001, adjusting for all facility-level covariates).

**Figure 2:**
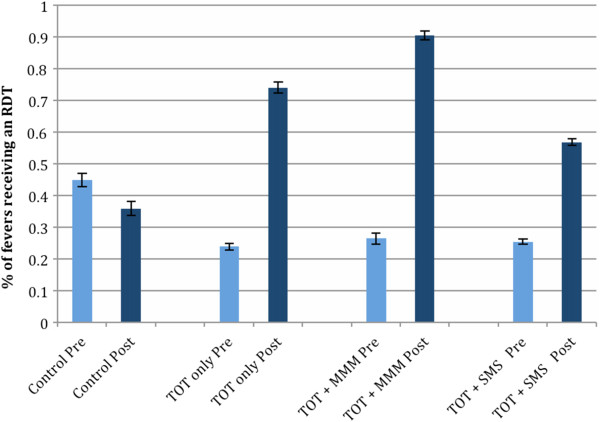
**Proportion of fevers tested for malaria in four districts of the Kavango Region, Namibia, before and after implementation of three types of training interventions.**

Controlling for facility-level covariates, significant increases were observed within all three intervention districts compared with the control district (all p < 0.02), although the increase in Andara, where TOT was accompanied by mentorship, was greater than in either the TOT-only district of Nankudu (Z = 2.28, p = 0.023) or Rundu, the district receiving TOT and SMS reminders (Z = 3.25, p = 0.001) after controlling for covariates. Mean monthly facility testing rates reached 91.6% in Andara, 75.9% in Nankudu, and 72.7% in Rundu, compared with 41.8% in Nyangana (Figure 2). Changes in monthly testing rates were not significantly greater in health facilities receiving SMS messages (Z = −1.21, p = 0.228) compared to the other intervention districts.

### Appropriate ACT use

In the six months prior to intervention, 1,046 patients in the intervention districts were inappropriately reported as receiving ACT after a negative test and 1,343 following only clinical diagnosis. After intervention, these numbers were reduced dramatically to only 26 and three, respectively. A total of 83 patients were reported to have received ACT at health facilities in intervention districts in the six months after the initiation of the intervention, a dramatic reduction from the 2,631 patients reported receiving ACT in the same six months during the prior year.

**Figure 3:**
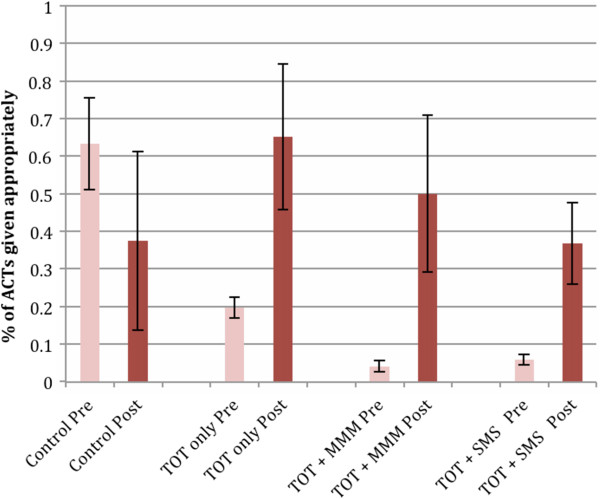
**Proportion of ACTs administered appropriately, in four districts of the Kavango Region, Namibia, before and after implementation of three types of training interventions.**

Controlling for all health facility-level covariates, the fraction of patients with negative RDTs recorded as receiving an ACT declined significantly more in health facilities in intervention districts than in the control district (Z = −3.13, p = 0.002), from a mean monthly health facility average of 36.1% to 1.5%. Similarly, the fraction of febrile patients recorded as receiving an ACT without any test declined significantly more in intervention districts (Z = −2.87, p = 0.004), from a mean monthly average of 30.0% to 0.1%. Controlling for all covariates, the change in the fraction of ACT that was prescribed following a positive RDT increased significantly in the intervention districts compared with the control (Z = 2.67, p = 0.008) (Figure 2). Total inappropriate ACT use, defined as the fraction of ACT given to negative and untested patients, is also depicted in Figure 3. Declines in inappropriate ACT use were significantly greater in the MMM district than in the district receiving only TOT (Z = −2.38, p = 0.017), but no significant impact of the SMS messaging was observed beyond the impact of TOT alone (Z = −0.93, p = 0.353).

### Sustainability

The 15 month follow-up data demonstrated that testing rates remained high. Over the 15-month follow up period, 21,980 out of 29,826 fevers (74%) were tested in the intervention districts, and no ACT was recorded as being prescribed without an RDT being administered first. Out of a total of 17,276 negative RDTs performed, only 11 ACT courses were prescribed to RDT negative patients; one from Andara district and 8 from Rundu district. 1220 out of 1236 RDT positive patients (98%) were correctly treated with ACT.

## Discussion

The training interventions described here resulted in substantial changes in the government of Namibia’s perception of its overall malaria burden and the distribution of malaria cases in the country. In 2011, Namibia reported a disproportionately high number of cases in the Kavango region. The results of this pilot suggest the great majority were due to misdiagnosis, resulting in a skewed understanding of the true burden of malaria and preventing accurate targeting of interventions to where they are truly needed. Improved diagnosis seems likely to have been a primary contributor of the national reduction in malaria from 14,401 reported cases in 2011 to only 2,882 cases in 2012, as the region of Kavango was the highest contributor to malaria cases in the country. The results of this investigation, which demonstrate dramatically improved testing rates with RDTs and declines in inappropriate prescription of ACT, led to national adoption of the enhanced training model and curriculum by the MoHSS and NVDCP, and training expansion to the 12 remaining regions during in the first quarter of 2013.

Even though the trainings were highly rated by the HCWs, some HCWs doubted the importance of understanding malaria because they claimed never to see it. Reinforcing and maintaining skills and awareness among HCWs for malaria diagnosis as it increasingly becomes a disappearing disease will be critical to ensuring elimination is achieved and maintained. An important focus of the new training format was on how to manage RDT-negative cases. In the post-intervention test, 92% of participants answered that “the healthcare worker should reassess the patient’s signs and symptoms for differential diagnosis,” compared to only 54% in the pre-tests. The substantial decrease in ACT treatment following negative tests corroborates that this message was understood.

Results suggest that following up initial trainings with supporting interventions may be useful for increasing adherence to case management guidelines, though more research will be needed to fully elucidate the optimal methods for doing so. Health facilities in the district that received MMMs in addition to the TOT saw the greatest improvement in HCW performance according to both the fraction of fevers tested and the fraction of ACT used appropriately, though this result is based on performance in a single district and must be replicated to see if they hold true elsewhere. However, it suggests that having HCWs interact as a cohesive social unit using multifaceted learning interventions [5] may result in correlated behaviors [9]. Interventions based on peer support rely on the role that peers’ judgment and beliefs play in an individual’s evaluation and interpretation of new information, and use the influence and pressure of persons in the target practitioners’ social network to affect individual performance [9]. The challenges of giving additional mentorship duties to key personnel who may already have many responsibilities and the potential cost implications of this intensive, onsite intervention need to be taken into account when considering national scale up. Mentors must have the capacity to manage multiple duties, and resources must be available to support the logistical and operational requirements of this intervention; enhanced trainings alone achieved similar results with lower resource costs.

During the six-month study period, SMS reminders did not result in improved case management beyond the TOT alone, and health facilities receiving the reminders had on average the lowest improvement of the three intervention districts. This result was likely influenced by the fact that Rundu district has the highest number of facilities compared to all the other study districts, as well as a large HCW turn-over. Over the course of the study period, many new HCWs were introduced to the health system and thus were not included in the original messaging roster. Revising the HCW roster and sending SMS reminders for an additional six months could have minimized the HCW turn over effects. However, proper case management remained consistent in the SMS district during the 15 month follow up. This was encouraging and favors the perception that SMS reminders may result in sustained effect of the intervention [27] over time and that using SMS reminders as a low-cost strategy to maintain practices between training sessions could be a possible supportive practice in Namibia and future exploration in the utility of SMS reminders should be explored.

This evaluation of Namibia’s case management trainings had several limitations. Although at low levels and independent from HCW case management, the omission of micrscopy data may affect results. Rates of RDT use could be skewed if there were changes in microscopy confirmation rates, especially in the Rundu district where the regional referral hospital is located. Another limitation is the register data collection process. Only six months of data were recorded directly from health registers due to limited resources and there was no way to confirm whether the registers completely represented what happened during the patient visit; it had to be assumed the HCWs recorded the actual diagnosis and treatment that occurred and not just what the HCWs knew was expected from them. The only validation used was a comparison on the number of RDTs and ACT that were on stock cards and what was recorded on the register. These were found to be accurate, as the HCWs are aware there would be diciplinary action if there were discrepancies found. NVDCP weekly surveillance data observed during the following 15 months after the study concluded provided a means of ensuring that practices did not decline rapidly following the cessation of the study, but this information was not available at the health facility level. In addition, control selection was not random in this investigation. Nyangana district had shown to be performing better than other districts in the region, and it was decided in collaboration with the NVDCP to target interventions to the districts with poorer case management practices to ensure public health benefit. In addition, although interventions were randomly assigned to the three districts, structural differences between the three areas (such as a different group of government personnel overseeing all health facilities within each district) may influence the observed results. Results may also vary since the districts have different numbers of health facility and staff turnover, meaning implementation of these interventions in different settings may produce different results. Despite these challenges inherent to non-randomized studies, the health system and community lifestyles were quite similar across districts, and the enhanced TOT model was consistently successful across intervention districts.

## Conclusion

Namibia’s case management pilot project successfully strengthened diagnosis and treatment practices in Kavango and demonstrated the far-reaching effects of proper case management training in an elimination context. These types of approaches will be important for the growing number of low endemic countries looking for guidance on sustainable approaches to febrile illness management and those facing similar challenges with low uptake of RDTs and adherence to results. As malaria incidence continues to decrease in eliminating countries, there is a need for new tools to support HCWs to effectively manage the non-malarial febrile cases, which comprise the great majority of febrile illness in these regions [28–30]. Improved diagnosis will provide a more accurate understanding of the malaria burden and where it occurs, which will allow malaria programmes to more efficiently target limited resources. Doing so in Namibia will shift the focus from achieving universal coverage with vector control interventions [31] throughout the endemic north to more precisely targeting them to where cases are truly occurring, accelerating progress towards the country’s elimination goal.

## Electronic supplementary material

Additional file 1: **An excerpt from the Malaria Case Management TOT Facilitator Manual: descriptions on course structure, agenda, and activities added to the NVDCP TOT model.** (PDF 368 KB)
