# Supplementary material for: Strengthening malaria diagnosis and appropriate treatment in Namibia: a test of case management training interventions in Kavango Region
Source: Malar J. 2014 Dec 18;13:508. doi: 10.1186/1475-2875-13-508 (PMC4301656; doi:10.1186/1475-2875-13-508)
Supplement: Supplementary file 1 — Additional file 1: An excerpt from the Malaria Case Management TOT Facilitator Manual: descriptions on course structure, agenda, and activities added to the NVDCP TOT model. (PDF 368 KB) [file 12936_2014_3639_MOESM1_ESM.pdf]

Additional file 1. An excerpt from the Malaria Case Management TOT Facilitator Manual: descriptions on course structure, agenda, and activities added to the NVDCP TOT model.

1A. Participant course structure

The course is designed following the normal provider - patient interaction process starting with looking for signs and symptoms of uncomplicated and complicated malaria and ending with the weekly routine surveillance of cases by the health care provider.

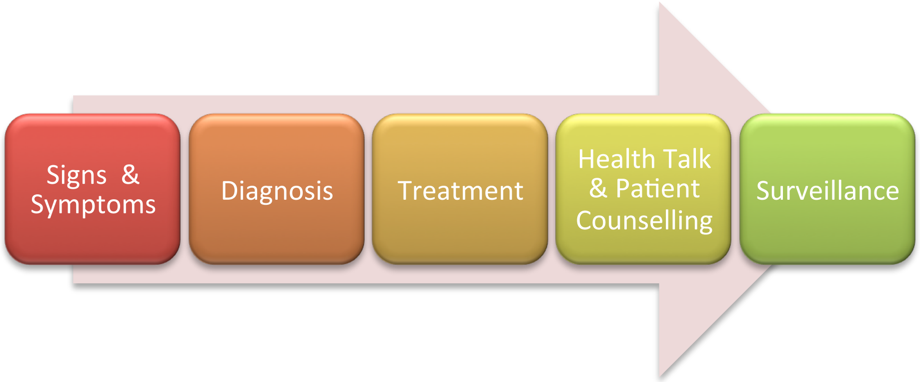

Module	Description
**Course Introduction**	This module provides background information about the overall objective, structure and content of the course.
**Module 1: Malaria Elimination**	In this module the concept 'Elimination' and Namibia's vision and strategies to achieve a malaria free nation are described.
**Module 2: Life cycle of Malaria**	The life cycle of malaria in a human body and in the mosquito are briefly refreshed.
**Module 3: Signs & Symptoms**	The clinical features of uncomplicated and severe & complicated malaria are described in this module.
**Module 4: Diagnosis & Rapid DiagnosticTest**	In this module the importance of testing and the use of the RDT test are discussed.
**Module 5: Differential Diagnosis for Fever**	The importance of applying differential treatment to negative test cases is presented i this module.
**Module 6: Malaria Treatment**	The treatment of uncomplicated and severe complicated cases, and the importance of adherence are outlined in this chapter.
**Module 7: Health Talk & Patient Counselling**	How to effectively educate patients and care givers about a number of key messages
**Module 8: Surveillance**	The importance of weekly routine surveillance and the use of the reporting format are explained in detail in this module.
**My Personal Action Plan**	Finally you can write down your key learning points and compile your action plan to help combat apply the learning in your day to day practice.

1B. Description of Enhanced Training Workshop Activities

Day 1	Activity name	Description
1	**Mosquito clap**	**RECOMMENDED TIME: Exercise 5 minutes**<br>The facilitator interacts with participants asking them the following questions:<br>▪ Do you have many mosquito's in this area?<br>▪ Imagine you are in a room pitch dark and you hear the mosquito....<br>▪ What will you do first? (swat, swat, swat the mosquito)<br>▪ Then think you got it, what do you do? (clap, clap, clap the mosquito)<br>▪ Then you want to make sure it's dead so you jump out of bed and what do you do? (stomp, stomp, stomp the mosquito)<br>▪ Repeat with lots of body movement
2	**60 seconds with**	**RECOMMENDED TIME: Exercise 25 -30 minutes**<br>Participants are asked to introduce themselves in 60 seconds. Ask participants to tell each other their name, where they work and mention a news item about themselves! The news item should be something new that not many people know about them or about what they are planning to do or recently have done.<br><br>Alternative 1: You can have participants introduce each other in 60 seconds so instead of the interviewee saying who s/he is the interviewer does the talking. (good alternative to practice listening skills, questioning skills....)<br><br>Alternative 2: Participants are free to interrogate each other on anything that comes to mind. The objective of the exercise is to get to know as much as possible about your colleague in 60 seconds. When the time is up everyone has to introduce their colleague.<br><br>TIP: If you are pressed for time you can change the exercise to 30 seconds!
3	**Gallery walk**	**RECOMMENDED TIME: Exercise 15 minutes + 15 minutes debrief**<br>Put up 4 flipchart papers in the four corners of the room and write the following questions on the flipcharts:<br>**1. What are your learning expectations?**<br>**2. What are your ground rules?**<br>**3. What challenges do you experience in the area of malaria case management?**<br>**4. What are frequently asked questions you often get from patients/colleagues?**<br>▪ Ask participants to split up in four groups (you can also allocate people to groups), each group stand up and goes to one of the flipcharts.<br>▪ Give each group a different colour marker (red, blue, green, black) in this way you can identify which group entered which submissions.<br>▪ Have participants answer the question they have on their flip chart within 2 minutes. Tell them to be very specific and clear especially on question 2, avoid vague learning expectations.<br>When the time is up, each group has to move clockwise to the next flip chart and first read what the previous group has written (1 minute) before adding their points (2minute). When the 2 minutes are over participants are asked to rotate (encourage them to run or dance if you have music to the next question gives a fun and competitive feeling to the exercise). After about 12 minutes all participants have answered the 4 questions and now it's time to ask a representative of each group to read out the submissions made by all the groups. So the group that started at question 1 debriefs question 1, the group that started at question 2 debriefs question 2 etc<br>After each question has been debriefed it is important that you provide feedback on each question, especially on the learning expectations. On questions 3 and 4 it's good to just briefly take note of the issues mentioned and state that you will address these during the workshop.
4	**Learning tree**	**RECOMMENDED TIME: Exercise 2 minutes + 3 minutes debrief**<br>Participants are asked to complete the learning tree in their manual after they have completed the pre-test. This will give the facilitator an idea of where participants feel they are in their learning journey. Ask all participants to say which figure they have selected and why.

Day 1	Activity name	Description
		Alternative: Use a laminated poster and give participants white board markers. Have everyone write their initial next to the figure on the tree that best represents how they feel at the moment in the area of malaria case management.
5	**Watch video clip**	**RECOMMENDED TIME: Watch 3 minutes + 2 minutes debrief**<br>Participants watch a short 3-4 min introductory video clip that illustrates the Malaria situation in the world and the what Malaria control and elimination entails.
6	**Count on Me**	Have each person think about the question raised: Who is responsible for elimination? Ask participants to share a key quality they possess that is needed in wiping out the disease. Have participant complete this sentence:<br><br>*Count on me....to wipe out malaria in Namibia by 2020!*<br><br>(write the sentence on a flip chart and asks them to say what good quality they possess to help wipe out malaria. (e.g. integrity, hard work, positive attitude, knowledge, etc.)
7	**Human Life Cycle**	**RECOMMENDED TIME: Exercise 10 minutes + 5 minutes debrief**<br>Preparation: Write on A4 white paper the 6 stages the life cycle in a human body.<br><br>Split the group in two groups. Give each group a ball of yarn (about 20 meters, split the one ball provided into two balls). Ask 6 participants in each group to stick one of the 6 stages on their chest. Make sure that everyone can clearly read them:<br>1. Infected mosquito<br>2. Bitten person<br>3. Liver<br>4. Bloodstream<br>5. Burst red blood cells<br>6. Fever<br>The remaining participants act as observers and can join the circle. They can be consulted when a participant is unsure about the next step.<br>Star the exercise by asking the group to say where the life cycle of malaria starts. The person they point to should then start with the exercise. This person has to call out what s/he has written on their chest and say: E.g. “I the infected mosquito need a bitten person to survive, because...”. S/he then throws the twine to the person who was called out. This person in turn says who s/he would need to survive till all stages have been completed.<br>Debrief the exercise by asking the observers where they think it went well and where things were said incorrectly. As a facilitator you should also provide feedback about the process.<br><br>TIP: Each facilitator should assist with a group. Ensure that participants are standing a bit mixed in terms of the stages, so that in the end a nice network is created by the yarn that is thrown around the circle.<br><br>Use the outcomes of the exercise to provide additional information with the slides.
8	**Body Mapping**	**RECOMMENDED TIME: Exercise 10 minutes + 10 minutes debrief**<br><br>Split the group in two groups. Have each group put 2-3 flipcharts on the floor. The flipcharts should be stuck together with masking tape make it one long piece of paper about 2 meters long (to save time it would be good to already prepare this for the groups). Each group should assign a volunteer who will lie on the floor and have his/her body shape drawn with a tick marker. When done participants have to use the body to then indicate with a green marker the parts that will show signs and symptoms of uncomplicated malaria and in red the signs and symptoms of complicated. At the end of their group work the teams have to present and compare outcomes. Ask participants:<br>▪ Is there anything you would like to add?<br>▪ Are there any surprises?<br>Use the outcomes of the exercise to highlight the key issues in the module.
9	**Practicum**	**RECOMMENDED TIME: Exercise 35 minutes + 10 minutes plenary debrief**<br>The practicum will consist of a number of small exercises:<br>A. Show some test results and ask participants to interpret them.<br>B. Finger pricking. Ask participants to prick each other’s fingers following the instructions given by the facilitator. Remind participants to also consult the RDT

Day 1	Activity name	Description
		Standard Operation Procedure for further information.
10	**Ball throw B's & C's**	**RECOMMENDED TIME: 5 minutes**<br><br>This exercise is meant to get some feedback from participants about the workshop. Ask participants to stand in a circle. Everyone will get a chance to say something about how the course has benefited (B) them so far or a concern (C) they have. *Example: My benefit is that I have learnt more about my role in elimination; a concern I have is whether we will be able to finish the workshop on time tomorrow.* Hereafter you have to throw the ball to any other person in the room. Tell participants that they do not necessarily have to share a concern but it's nice to hear them if people have any.<br><br>TIP: make sure there is sufficient room to play this game, alternatively go outside.
11	**People to people**	**RECOMMENDED TIME: 5 minutes**<br>For this icebreaker you need uneven number of participants. Ask all participants to hold hand with a partner. You as the facilitator should stand alone. Tell participants that you will call out instructions they have to carry out with their partner. When you say People to people everyone has to run around quickly and get another partner. No one is allowed to just take the person standing next to them. The facilitator now also runs to get a partner as fast as possible. The person left standing alone has to now say a number of instructions and end with the saying people to people to create again a great chaos in the room. Some instructions that are called out are:<br>▪ Toe to toe<br>▪ Head to knee<br>▪ Back to back<br>▪ Finger to finger<br>▪ Hip to bum<br>▪ Etc.
12	**Newspaper headlines**	**RECOMMENDED TIME: Exercise 10 minutes + 10 minutes debrief**<br>This exercise can be used to recap the learning of the previous day. Ask participants to pretend they are a news paper reporter for a local news paper. They should write down a few key headings of news items which capture the key learning points of the previous day. Split the group in 4 groups and give each group some a pack of pens. Encourage participants to also make drawings. Upon completion each group has to report back.
13	**Case study<br>Differential Diagnosis**	**RECOMMENDED TIME: Exercise 15 minutes + 15 minutes debrief**<br><br>In section 5.4 in the facilitator manual there are 5 case studies on differential diagnosis. Create 5 groups and assign each a different case study. Alternatively you can select 1 case study and have the entire group work on it. Give participants the time to read and answer the case study for themselves before they discuss it in smaller groups or plenary. Upon completion review the key learning points (see answers to case studies with take home messages) and enhance with presentation slides on the topic.
14	**Improvised role plays for:**<br><br>**Treatment of (un)complicated cases**<br><br>**PLUS**<br><br>**Patient counselling**	**RECOMMENDED TIME: Exercise 20 minutes (role plays) + 10 minutes plenary debrief**<br><br><u>Do this exercise after you have presented the theory!</u><br><br>Create small groups of 3 people. If you have an even number then 1 group will have 4 participants. Ask participants to role play in the room (spread them around) a patient counselling scenario. They should take a situation they find difficult to handle. Actor 1 = Patient; Actor 2= Health care worker, the third and /forth person = observer(s). The role of the observer is to give feedback to the health care worker. S/he should point out what went well and what could be improved. Prior doing this, the observer should allow the health care worker to first critique him/herself!<br><br>If time allows have participants switch roles so that everyone get a chance to be a health care worker.<br><br>When participants are busy role playing facilitators should "float" around the room as super observers trying to get a sense of the interactions and feedback given.

Day 1	Activity name	Description
		Upon completion of the role plays debrief the learning and reinforce the key learning points. Debriefing questions:<br>▪ How was it to do this exercise?<br>▪ What were issues everyone struggled with?<br><br>TIP: Demonstrate a role play plenary before you start with a participant as a patient and the co-facilitator as the observer.
15	**Surveillance report**	**RECOMMENDED TIME: Exercise 20 minutes + 10 minutes plenary debrief**<br><br><u>Do this exercise after you have presented the theory!</u><br><br>Give everyone a copy of a case study data with an uncompleted surveillance report for the corresponding situation. Split the participants into groups of 2 (or 3 if uneven numbers). Ask everyone to fill out the form and to note any issues or questions. Upon completion, the facilitator will go through each column and ask the groups what the right answers should be. If there are any disagreements between the groups' answers, spend time on that column.<br><br>Hereafter you can debrief the exercise in plenary. Handout a copy of the correct surveillance report for the case study.
16	**Post test & Learning tree**	**RECOMMENDED TIME: Exercise 20 minutes + 10 minutes plenary debrief**<br>Give everyone a copy of the post test and 20 minutes to complete the answers. Upon completion they can go to the learning tree in their manual and assess themself post the workshop. Where are you now in the learning tree? Have you moved upwards, downwards, sideways or are still at the same point where you were yesterday when we started the training?<br><br>TIP: If you have time do a plenary debrief of the learning tree.
